# Lesion detection in demoscopy images with novel density-based and active contour approaches 

**DOI:** 10.1186/1471-2105-11-S6-S23

**Published:** 2010-10-07

**Authors:** Mutlu Mete, Nikolay Metodiev Sirakov

**Affiliations:** 1 Department of Computer Science, Texas A&M University–Commerce, Commerce, Texas, USA; 2 Department of Mathematics, Texas A&M University–Commerce, Commerce, Texas, USA

## Abstract

**Background:**

Dermoscopy is one of the major imaging modalities used in the diagnosis of melanoma and other pigmented skin lesions. Automated assessment tools for dermoscopy images have become an important field of research mainly because of inter- and intra-observer variations in human interpretation. One of the most important steps in dermoscopy image analysis is the detection of lesion borders, since many other features, such as asymmetry, border irregularity, and abrupt border cutoff, rely on the boundary of the lesion.

**Results:**

To automate the process of delineating the lesions, we employed Active Contour Model (ACM) and boundary-driven density-based clustering (BD-DBSCAN) algorithms on 50 dermoscopy images, which also have ground truths to be used for quantitative comparison. We have observed that ACM and BD-DBSCAN have the same border error of 6.6% on all images. To address noisy images, BD-DBSCAN can perform better delineation than ACM. However, when used with optimum parameters, ACM outperforms BD-DBSCAN, since ACM has a higher recall ratio.

**Conclusion:**

We successfully proposed two new frameworks to delineate suspicious lesions with i) an ACM integrated approach with sharpening and ii) a fast boundary-driven density-based clustering technique. ACM shrinks a curve toward the boundary of the lesion. To guide the evolution, the model employs the exact solution [27] of a specific form of the Geometric Heat Partial Differential Equation [28]. To make ACM advance through noisy images, an improvement of the model’s boundary condition is under consideration. BD-DBSCAN improves regular density-based algorithm to select query points intelligently.

## Introduction

Melanoma is the fifth most common malignancy in the United States. Malignant melanoma, the most deadly form of skin cancer, is one of the most rapidly increasing cancers in the world. An estimated amount of 8,441 deaths out of 68,720 cases were recorded in the United States in 2009 [1]. Early diagnosis is particularly important, since melanoma can be cured with a simple excision if detected early.

Dermoscopy, a non-invasive skin imaging technique, has become one of the most important instruments in the diagnosis of melanoma and other pigmented skin lesions. It involves optical magnification of the region-of-interest, which makes subsurface structures more easily visible compared to what can be seen via the naked-eye [2]. This, in turn, improves screening characteristics and provides greater differentiation between difficult lesions such as pigmented Spitz nevi and small, clinically equivocal lesions [3]. However, it has also been demonstrated that dermoscopy may actually lower the diagnostic accuracy in the hands of inexperienced dermatologists [4]. Therefore, new frameworks for the understanding of computerized images are needed to minimize the diagnostic errors that result from the difficulty and subjectivity of visual interpretation [5][6].

For melanoma investigation, delineation of the region-of-interest is the first and most important step in the computerized analysis of skin lesion images for many reasons. First of all, the border structure provides important information for accurate diagnosis. Asymmetry, border irregularity, and abrupt border cutoff are just a few of the clinical features calculated based on the border lesion. Furthermore, the extraction of other important clinical indicators such as atypical pigment networks, globules, and blue-white areas (irregular, structureless areas of confluent blue pigmentation with an overlying white ground-glass film) critically depends on the border detection [7][8]. 

In the literature, many algorithms were proposed regarding border detection in dermoscopy images. These include the PCT/median cut algorithm [9], adaptive thresholding in the first image plane of the PCT [10], thresholding in the blue image plane[11], k-means clustering [12], split-and-merge [9][13], a segmentation technique based on a Markov random field (MRF) image model [14], and a non-linear diffusion technique [12]. Furthermore Schmid et al. [15] proposed an algorithm based on color clustering. In their study, a two-dimensional histogram is calculated first from the first two principal components of CIE L*u*v* color space. The histogram is then smoothed, and initial cluster centers are obtained from the peaks using a perceptron classifier. In the final step, the image of the lesion is segmented using a modified version of the fuzzy C-means clustering algorithm. Gao et al. [12] created two methods: one based on stabilized inverse diffusion equations, a form of non-linear diffusion, and another one based on Markov random fields in which the model parameters are estimated using the mean field theory. 

The active contour approach was developed in late eighties by the work of Kass, Witkin, Terzopoulos [16], Osher and Sethian [17] and quickly became very popular, providing excellent results in almost all areas of its application. One such area is biomedical image analysis with a number of strong methods and algorithms. Without underestimating the contributions of the other works, Acton and Ray wrote one important book that analyzes the advantages and limitations of the active contour methodology [18]. 

From the angle of cluster boundaries, Lee and Castro [19] introduced a new algorithm of polygonization based on the boundaries of resulting point clusters. Recently, Nosovskiy et al. [20] used an adaptive function approach to find the boundary of a cluster in order to infer accurate boundaries between close neighboring clusters. These two works principally focus on the boundaries of finalized data groups (clusters), which is not the case for our present work.

In this study, we introduce a new framework in which a novel, fast, and accurate ACM is integrated with a filtering approach, as well as a new data mining approach to be used in boundary detection. Both methods are compared regarding their ability to define the boundaries of skin lesions. In short, ACM starts with a preprocessing step by denoising the input image and increasing the homogeneity of the background. This is the only preprocessing step taken before the active contour is run. On the other side, the boundary-driven density-based algorithm (BD-DBSCAN) [21] requires a binary (thresholded) image and highlights significant regions of the lesion. In Table 1 we reported recall, precision, accuracy, and border error [9] for 50 images. One may tell from Table 1 that average border errors of both methods are found to be equal. 

**Table 1:** Border error, precision and recall measures for images in the dataset.

		ACM			BD-DBSCAN	
**Img. ID**	**Border Error**	**Precision**	**Recall**	**Border Error**	**Precision**	**Recall**

1	13.3%	0.76	0.94	**8.2%**	0.98	0.79
2	**6.7%**	0.92	0.91	8.0%	0.93	0.86
3	**3.8%**	0.86	0.96	4.9%	0.89	0.85
4	8.0%	0.93	0.84	**6.2%**	1.00	0.82
5	**3.9%**	0.92	0.95	4.6%	1.00	0.83
6	11.0%	0.75	0.99	**3.9%**	0.96	0.91
7	22.2%	0.95	0.27	**3.2%**	1.00	0.87
8	6.2%	0.80	0.90	**3.4%**	1.00	0.82
9	3.8%	0.87	0.98	**2.2%**	1.00	0.91
10	2.8%	0.79	0.99	**0.9%**	1.00	0.91
11	**5.7%**	0.99	0.66	6.5%	1.00	0.61
12	**10.0%**	1.00	0.79	14.8%	1.00	0.70
13	**3.1%**	1.00	0.82	5.9%	1.00	0.67
14	**4.6%**	1.00	0.84	6.8%	1.00	0.76
15	7.6%	1.00	0.60	**6.0%**	1.00	0.67
16	**4.4%**	1.00	0.80	6.4%	1.00	0.71
17	**4.5%**	0.99	0.89	8.8%	1.00	0.78
18	**5.3%**	0.97	0.91	12.6%	1.00	0.73
19	**5.5%**	0.99	0.85	8.6%	1.00	0.76
20	**5.0%**	1.00	0.82	5.7%	1.00	0.79
21	**8.2%**	1.00	0.76	9.0%	1.00	0.74
22	**4.5%**	0.99	0.80	8.0%	1.00	0.65
23	**5.3%**	0.99	0.87	10.6%	1.00	0.75
24	**6.6%**	0.99	0.85	11.3%	1.00	0.74
25	**7.0%**	1.00	0.85	10.8%	1.00	0.77
26	**2.3%**	0.98	0.96	4.2%	1.00	0.88
27	**3.2%**	0.99	0.89	4.0%	1.00	0.85
28	**7.9%**	1.00	0.71	8.0%	1.00	0.71
29	**4.7%**	0.99	0.84	8.0%	1.00	0.73
30	**1.5%**	0.97	0.96	3.2%	1.00	0.85
31	**5.3%**	1.00	0.81	7.3%	1.00	0.74
32	**2.5%**	0.98	0.90	3.6%	1.00	0.84
33	11.4%	0.68	1.00	**3.0%**	1.00	0.87
34	**5.6%**	1.00	0.83	10.9%	1.00	0.68
35	**3.9%**	0.99	0.88	7.4%	1.00	0.78
36	**3.6%**	0.93	0.98	4.2%	1.00	0.88
37	**14.6%**	1.00	0.61	14.9%	1.00	0.60
38	**5.3%**	1.00	0.87	9.4%	1.00	0.77
39	**3.2%**	0.91	0.92	4.6%	1.00	0.75
40	**1.2%**	0.98	0.94	2.9%	1.00	0.81
41	8.8%	0.79	0.76	**6.5%**	0.90	0.74
42	**5.1%**	1.00	0.77	5.8%	1.00	0.74
43	**4.5%**	0.99	0.82	5.6%	1.00	0.77
44	**1.7%**	0.97	0.92	2.9%	1.00	0.82
45	**7.4%**	0.99	0.74	8.3%	0.89	0.79
46	**4.6%**	0.95	0.92	6.3%	0.98	0.83
47	30.8%	0.39	1.00	**3.2%**	1.00	0.79
48	**0.7%**	0.99	0.95	2.4%	1.00	0.79
49	16.9%	0.53	0.96	**4.6%**	1.00	0.74
50	**5.0%**	0.98	0.84	8.8%	1.00	0.71

**Min**	**0.7%**	**0.39**	**0.27**	**0.9%**	**0.89**	**0.60**
**Max**	**30.8%**	**1.00**	**1.00**	**14.9%**	**1.00**	**0.91**
**Mean (µ)**	**6.6%**	**0.93**	**0.85**	**6.6%**	**0.99**	**0.78**
**Stdev (σ)**	**0.05%**	**0.12**	**0.13**	**0.03%**	**0.03**	**0.08**

## Results and discussion

### Dataset

As we mentioned above, the two methods are tested on a set of 50 dermoscopy images obtained from the Edra Interactive Atlas of Dermoscopy [2]. These are 24-bit RGB color images with dimensions ranging from 577 × 397 pixels to 1921 × 1285 pixels. The benign lesions include nevocellular nevi and dysplastic nevi.

### Preprocessing for BD-DBSCAN

**Figure 1:**
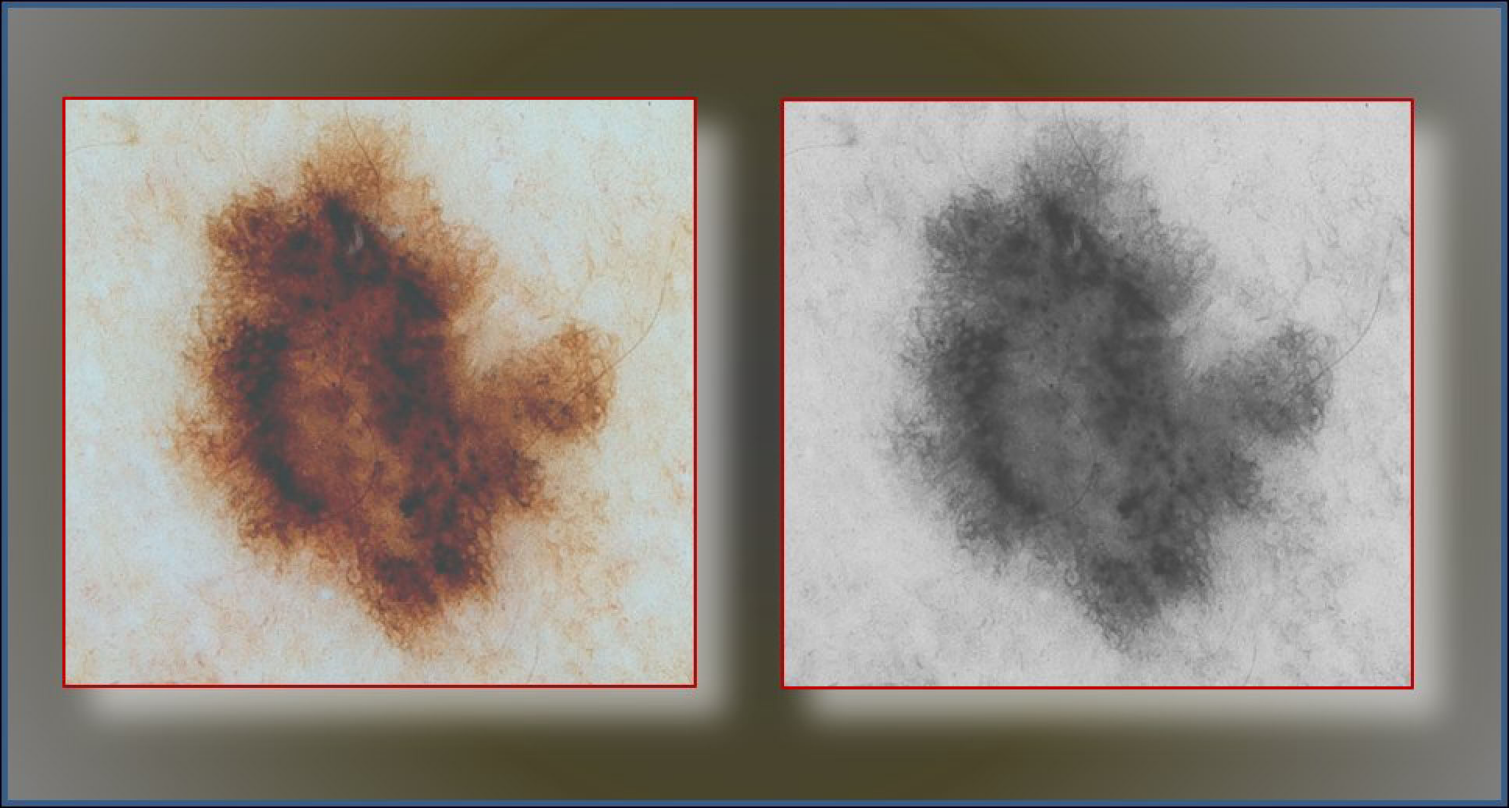
**Raw patient image (left) and grayscale representation of it (right).** Histogram-based thresholding methods mainly work in grayscale images. The range of values in (right) varies between 0-255.

The original dermoscopy dataset is obtained in 24-bit PNG format and includes three channels: red (R), green (G), and blue (B). To make use of a density-based algorithm, we first represent each image as one channel of luminance (gray scale). Toward this goal, for each RGB pixel, the lightness component of the HSL color space [22] is used: 

Lightness is the average of minimum and maximum color values of a pixel. Figure 1 depicts an original (RGB) image and its grayscale presentation obtained from the original one using the above formula. 

In the next step, a grayscale image is segmented with the intermeans algorithm, developed by Ridler and Calvard [23]. Actually, this is an iterative technique for choosing a threshold. Similar to many other segmentation algorithms, it takes the image histogram and initially assigns a threshold *T*. Compiling a histogram is an efficient and simple mean of representing an image as a one-dimensional array based on quantity of color samples. A histogram is the graphical representation of a resultant array using a bar chart or different visual modalities. Throughout this study, the histogram is denoted by y_0_, y_1_, y_2_, …, y_n_, where y_i_ is the number of pixels having gray-value i. The maximum value for the subscript i = {0, 1, …, n} is 255 for all images used in the present study. Thus the threshold T = {0, 1, …, n} is used to split the histogram into two groups. All pixel values less than or equal to T are assigned to one group, and those that are greater than T are assigned to the second group. 

### Intermeans segmentation

This iterative algorithm assumes that the image contains one object and a background region around it. This implies that each pixel comes from either an object or its background region. The algorithm starts with an initial guess for , where *j* and *k* are minimum and maximum pixel values of the current image. Then, it finds the means of the two classes defined by *T*, μ1 and μ2 respectively. 

**Figure 2:**
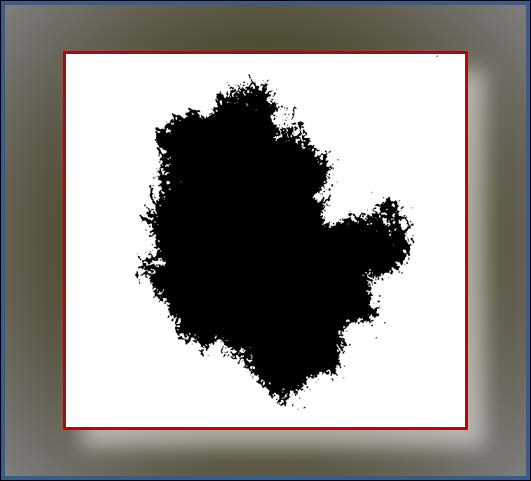
**A binary image generated by INTERMEANS thresholding on the grayscale image of Figure **1. This image is now ready for BD-DBSCAN algorithm, which considers each positive pixel a data point in 2D search space.

Having the initial means, the new *T_k_* is found by . The equality *T_k_* = *T_k_*_−1_ finalizes the search algorithm, and *T_k_* is used as the final threshold *T* for the current image. Applying threshold *T* to our dermoscopy images, we transform every image into a binary image. Figure 2 shows the binary outcome of this algorithm applied on the grayscale image in Figure 1. Note that in the binary image shown in Figure 2, pixels in the foreground (skin lesion, shown in dark) and those in the background (normal skin, shown in white) will be referred as positive and negative, respectively. Having a thresholded (binary) image for each color image, we run BD-DBSCAN on the binary one with *R* of 5 and *MP* of 60. The roles of parameters *R* and *MP* of BD-DBSCAN will be explained in the methodology section of the study. The algorithm focuses only positive pixels, considering those data points to be clusters in 2D space.

### Preprocessing for ACM

**Figure 3:**
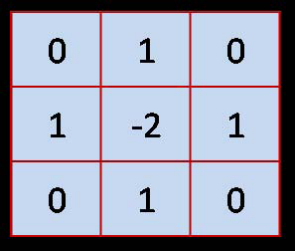
**The mask used to remove noise for ACM.** The optimum parameters in Eq. 1 and Eq. 2 give the mask. As highlighted in the discussion section, these parameters are to update for other frameworks. The size of the mask is fixed through this study.

**Figure 4:**
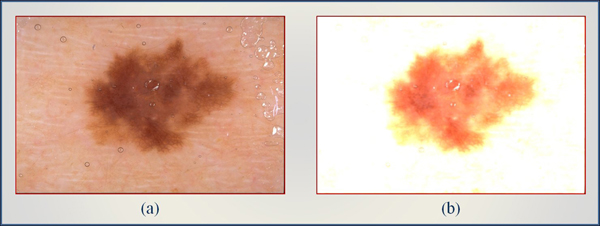
**The mask of Figure **3**  is applied to a raw dermoscopy image (a).** The process removed the noise in upper-right corner as shown in (b). The mask of Figure 3  is employed as denoising filter on current image. Also, most of the bubbles are disappeared on the left of the original image (a).

As discussed, ACM is combined with a filtering (high boost) approach to facilitate the convergence of the active contour. Thus, every colored image is treated with a mask defined by Eq. 1. Varying the coefficients (*A, B,* and* C*) in this equation produces different masks. The active contour is defined with Eqs. 3, 4, and 5, and its performance depends on two parameters *s* and *t*. To find optimal parameters, we took into account the set of images under consideration and the theoretical properties of the high boost and active contour approaches described in the section entitled Theoretical Derivation of Active Counter. Thus, a few preliminary experiments confirmed the expectation that *A=6, B=0,*and* C=1* will produce a mask (see Figure 3) from which the active contour will most benefit. The effect of this mask is shown in Figure 4. It is obvious that the background became uniform as the artifacts were removed, while the boundary of skin lesion was kept without change (Figure 4(a)). Note that the masked image, Figure 4(b), is also in RGB space, as is the original image. As for ACM, we used  points on the initial contour, where *P* is the perimeter and *s* is the arc length parameterization of the initial contour (see Eqs. 3 and 4). The time step *t* (the speed of convergence) of the approach was selected on the basis of the image size. The approach is coded in Java and used to perform the experiments shown in the Results section.

### Results

**Figure 5:**
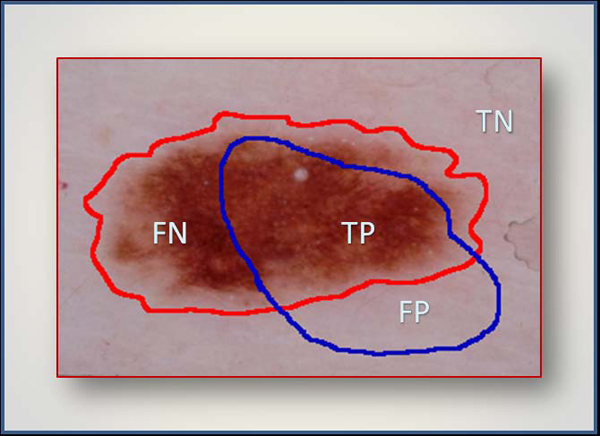
**Illustration of information components used in accuracy and error quantification.** Red and blue boundaries are drawn by a dermatologist and a non-expert −an automated framework, respectively. TP indicates correct lesion region found automatically. Similarly, TN shows healthy region (background) both manual and computer assessment agree on. FN and FP are labels for missed lesion and erroneous positive regions, respectively.

We evaluated border detection error and accuracy of ACM and BD-DBSCAN by comparing their results with dermatologist-drawn boundaries as a set of ground truths. Manual borders were obtained by selecting a number of points on the lesion border, connecting these points by a second-order B-spline, and finally filling the resulting closed curve [24]. Using the dermatologist- drawn borders, the automatic borders determined by the algorithms are compared using three quantitative error metrics: border error, precision, and recall. Border error was developed by Hance et al. [9] and given by

where *AutomaticBorder* is the binary image obtained with BD-DBSCAN or ACM. *ManualBorder* is the binary image as described above. Exclusive OR operator, ⊕, essentially emphasizes disagreement between target *(ManualBorder)* and predicted *(AutomaticBorder)* regions. Referring to information retrieval terminology, the nominator of the border error means summation of False Positive (FP) and False Negative (FN). The denominator is obtained by adding True Positive (TP) to False Negatives (FN). An illustrative example is given in Figure 5.

In addition to border error, we also reported precision (positive predictive value) and recall (sensitivity) for each experimental image in Table 1. Precision and recall are defined as   respectively. In addition, we can express border error with these widely used definitions, 

Note that border error and accuracy measurements run over number of pixels in particular regions. Analogously, *Area(.)* function returns the number of positive pixels in a thresholded image.

**Figure 6:**
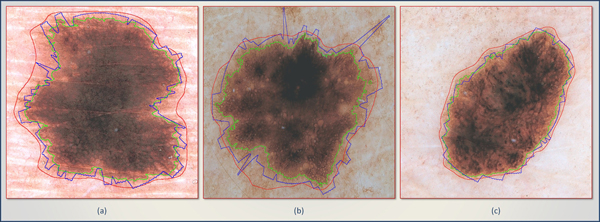
**Delineations of proposed frameworks are shown in three images.** Red, blue, and green indicate decision of dermatologist, region found by ACM, and BD-DBSCAN respectively. BD-DBSCAN tends to find small regions compared to ACM.

Table 1 gives minimum, maximum, mean, and standard deviation of border error, precision, and recall. It is observed that the results vary significantly across the images. Figure 6 shows three samples comparing automated framework and manual delineations on them. Quantitative accuracy measures of Figure 6(a),  6(b), and 6(c) are given with Image ID 14, 18, and 27 in Table 1. 

Both methods presented in this study give the same average border error of 6.6% in all 50 images, which is a very promising result in terms of a computer-aided framework. This means that, on average, given a dermoscopy image, either algorithm will identify 93.4% of the targeted skin lesions correctly. However, ACM’s standard deviation is slightly greater than that of BD-DBSCAN (0.05 > 0.03), indicating that BD-DBSCAN is more resistant to underlying variation in images. This is also confirmed when minimum and maximum values are compared. Although ACM’s minimum border error is less than that of BD-DBSCAN (0.7% < 0.9 %), this small difference is not seen when comparing maximum border errors (30.8% >> 14.8%). Statistically higher standard deviation and a few extreme values of boundary errors, such as 30.8% and 22.2%, cause ACM to have the same mean on results. However, a pairs-wise comparison shows that ACM performs better than BD-DBSCAN in 38 out of 50 cases. In Table 1, bold numbers in the boundary error column indicate better results in the corresponding images. 

We did not perform the t-test for this comparison because the major assumption of the t-test is not met within the results of ACM. The t-test requires normally distributed variables to compare. To check the normality of boundary errors, we used the Lilliefors test [25], which shows that the boundary errors of ACM is not normally distributed (p-value:0.001, critical value: 0.1245). Although BD-DBSCAN passed the normality test (p-value:0.3906, critical value:0.1245), the t-test would be inappropriate in this study.

### Discussion

Skin cancer is the one of most common malignancies in the United States and should be treated accurately by means other than manual delineation. The frameworks presented in this study play a key role in alleviating inter- and intra-variability in medical assessments. 

By its nature, ACM is more sensitive to noisy images. As seen in Figure 6(b), three spikes are caused by noisy pixels. BD-DBSCAN can also be negatively affected by noise, but, unlike ACM, the erroneous region is bounded locally. BD-DBSCAN usually finds a marginally shrunk version of the lesion, (Figure 6(a), 6(b), 6(c)) having several precision values of 1.00, as seen in many cases in Table 1. In a few cases, it also marked outer regions (FP) of skin lesions. The average ratio of precision implies that the regions found by BD-DBSCAN are usually smaller than those defined by ACM because of high means of precision (0.99 > 0.93).This observation is also examined in each of the three samples of Figure 6. In each of them, the green boundary (generated by BD-DBSCAN) is narrower than the blue, comparatively. On the other hand, recall rates on two groups suggest that ACM is more successful in finding more pixels of targeted skin lesion. BD-DBSCAN outperforms ACM only in these three images. 

Apparently, the region between red (drawn by the dermatologist) and green boundaries seems to be a major problem for BD-DBSCAN. Alternatively, it means that the manual border tolerates background errors (FP) in order to increase recall. Therefore, a dermatologist’s manual selection might not be accurate in delineating the exact region of lesion at a very fine level. Based on this assumption, the transition regions found between red and green boundaries would be expected consequences in this framework. This observation opens the door to the problem of intra-observer variability that needs more attention from researchers. It is suggested that the users of these frameworks look for various parameters that can be optimized for the underlying data. The mask obtained with Eq. 1 and Eq. 2 significantly affects the image in the first step. The small changes in parameters *t* and *s* are less effective inputs regarding the general design of ACM. Therefore, we will focus on newer techniques that lessen the negative impact of preprocessing in ACM. Similar to that of ACM, the preprocessing step of BD-DBSCAN can change delineation significantly. Other than a non-parametric histogram-based thresholding mechanism, a new set of binarization methods can be investigated. Parameters *R* and *MP* of BD-DBSCAN are less questionable in the context of this study, since they are good for each image once an agreement is reached.

In this study, we introduce and compare two frameworks based on the novel ACM and BD-DBSCAN in order to automatically detect skin lesions in dermoscopy images. A large number of active contour and level set algorithms are available in the field. A good survey is given in [18]. We have used the one presented in [27] because it has a larger capture rate, fewer arithmetic operations, better accuracy, but worse performance with noise when compared to the others. Similarly, BD-DBSCAN presents an innovative solution for fast calculation of lesion boundaries. Thanks to boundary definition of the cluster, it eliminates a huge number of region queries. The proper preprocessing steps are explained within each of the frameworks. Both of these algorithms have the same average boundary errors, 6.6%.

## Methodology

### Theoretical derivation of active counter

The present sections develop the theoretical fundament of the novel algorithm, described in the previous part of the paper, designed to automatically determine the boundary of lesions. As discussed in the Introduction, the new algorithm combines two approaches: the first one uses the so-called high boost filtering idea presented in [26]; the second one applies a version of the new ACM recently reported in [27].

The filtering approach is designed to facilitate the convergence of the active contour model, which could catch unnecessary objects or noise at the time of evolution. To avoid doing this, the image is processed in a way to eliminate such objects through sharpening and increasing the homogeneity of its background. One useful approach is to apply the high-boost approach to develop a new and useful un-sharpening mask [26]. 

The above idea is applied in the present study, and a new formulation of the framework is given hereafter. Assume the image is presented with the function *f(x,y),* where *(x,y)* gives the coordinate of a pixel whereas *f(x,y)* shows the gray level of this pixel. For simplicity’s sake,  we consider a single color channel in this presentation. In the case of a colored image, the work is extended to the three used channels.

Consider the following expression:

	(1)

where *A, B,* and* C* are integers, whereas

	(2)

is the sum of the directional derivatives in the directions of the vectors (±1, ±1). The last term of Eq. 1 represents the Laplacian of the image. 

**Figure 7:**
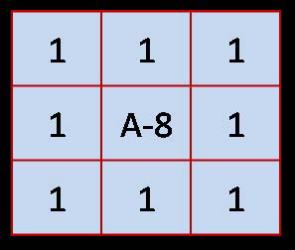
A **3 × 3 mask produced by Eq. 1 calculating the derivatives on two consecutive nodes.** This mask can be used as a template mask depending on only A of Eq. 1.

Using finite differences on two nodes, Eq. 1 and 2 generate the mask shown in Figure 7 if *B=1*, *C=1*. Calculating derivatives on higher numbers of nodes will produce masks with higher dimensions, but this work is not in the scope of this study. 

Now varying *A, B,* and* C* in Eq. 1, we could generate a mask with different entries. Each mask manipulates the image in a different manner. For example, if the sum of the mask’s entries is zero, the image will appear with a dark background, and the objects will have tiny white boundaries. Increasing the sum of the mask’s entries will sharpen the image, lighten the background, and make it more homogenous by erasing the small details. One potential disadvantage from a mask with a high sum of entries is that some light zones of the lesion may disappear as well. Using this knowledge, it is not difficult for a user to determine the right values of *A, B,* and *C* with respect to the given set of images.

**Figure 8:**
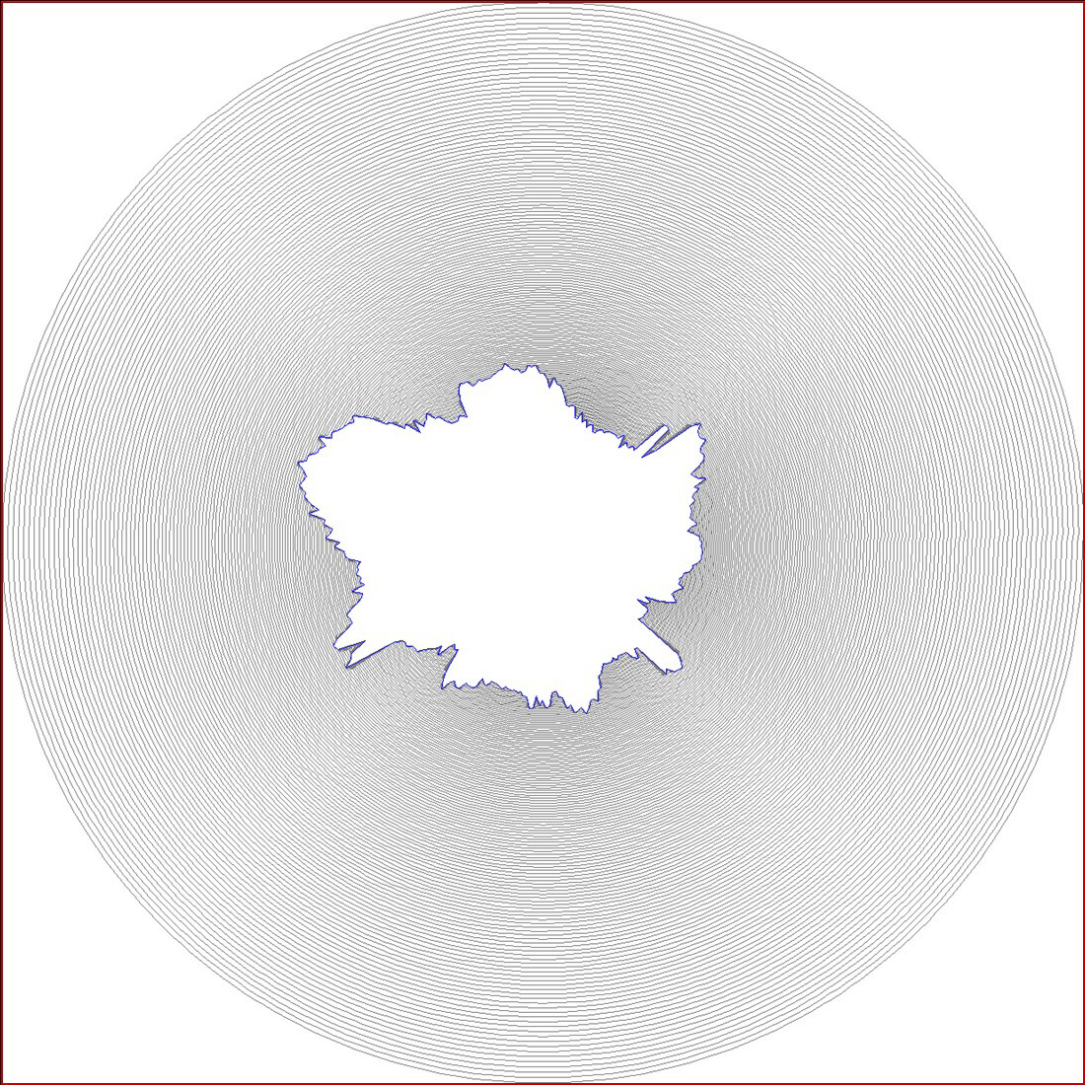
**A toy example showing family of curves obtained with *t=10* in Eq. 3.** When compared to Figure 9, it is noticeable that the step size in this family is small; thus, gives more dense structure.

**Figure 9:**
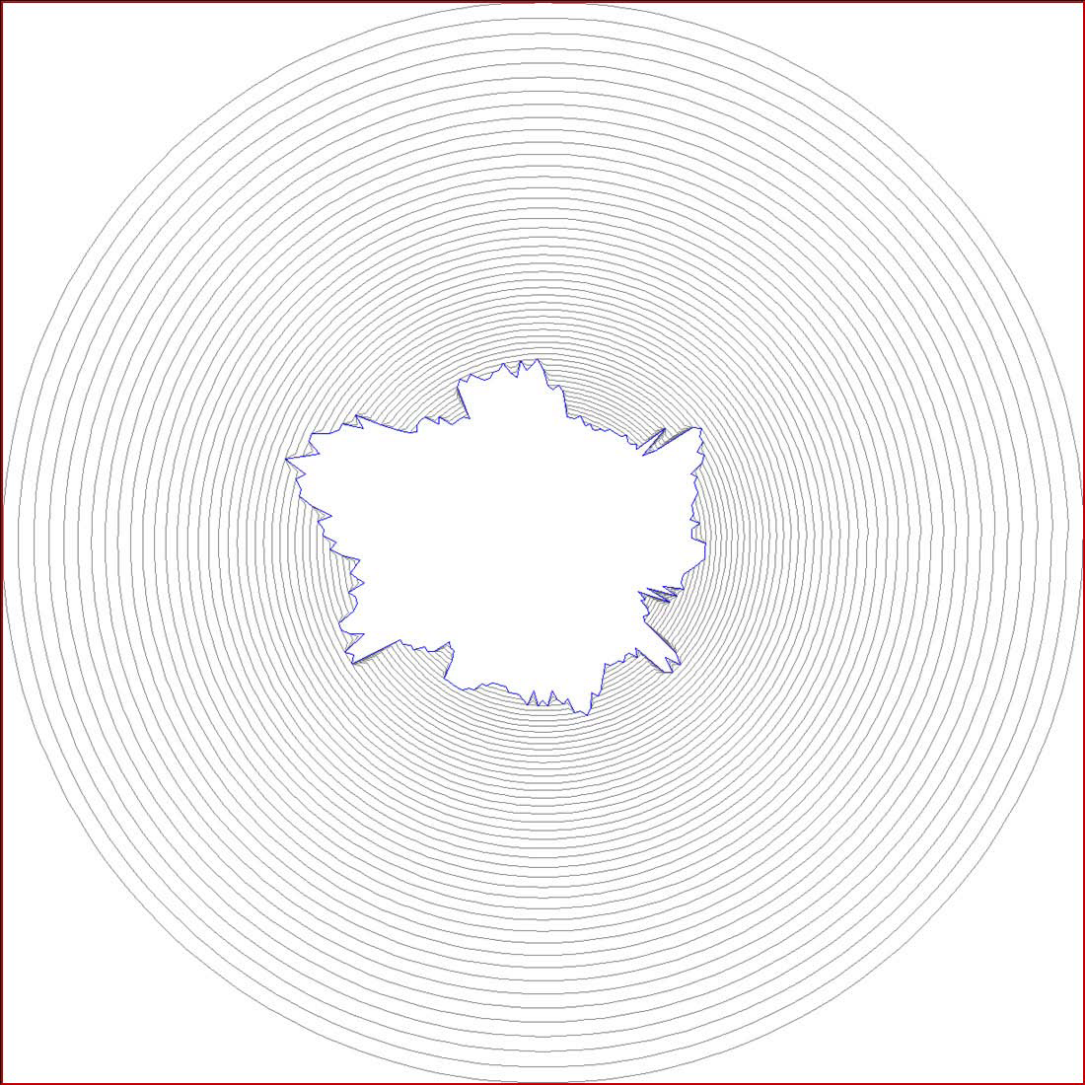
A toy example showing family of curves obtained with *t=30* in Eq. 3.

In the second stage of the present approach, an active contour based on the exact solution of the so-called Active Convex Hull Model (ACHM) [28] is applied to determine the boundaries of the lesions. The vector form of the solution, presented in [27], is given with the following equation:

	(3)

where *s* is a space parameter to define a particular contour, and *t* is a time parameter to define the family of the active contours and the speed of motion. Figure 8 and Figure 9 show an object and the family of curves evolving to its boundary. The time step used for each image was *t=*10 and *t=*30, respectively. 

Eq. 3, along with the following initial and boundary conditions, defines the active contour model used in the present paper. The initial condition:

 	(4)

where , *nc* denotes the number of columns of the image, whereas *nr* denotes the number of rows. The boundary conditions states that:

if  for *t>0.001* and s=s* then 				(5)

otherwise 					 				(6).

Eq. 5 states that, if the inequality is satisfied, the new value of the active contour at the point s* in time is set to be the same as the value at time *t*. If the inequality is not satisfied, the new value of the parametric function* r,* at time is calculated and used further by ACM.

The complexity of the calculation of this approach is in the order of where  The accuracy of boundary approximation is in the order of , where Δ*t* is a value given by the user and could be selected in a way to minimize the error of boundary delineation. 

### Boundary driven density based algorithm

Clustering, a major problem in the scope of unsupervised learning, deals with recognizing meaningful groups that include similar items. With the increase of digital data all around the world, more powerful tools are required to exploit piles of so-called useless datasets. Even though there is now substantial body of research on clustering, the constraints (e.g., efficiency and effectiveness) of current approaches require more practical algorithms. BD-DBSCAN demonstrates how the efficiency of prominent density-based clustering algorithm DBSCAN [29] is improved for skin lesion detection. 

The boundary-driven density-based algorithm is an intelligent technique that can be applied to any thresholded image to find the most represented objects(s) in the current scene. The rationale behind BD-DBSCAN is to evaluate pixels regarding their likelihood of expanding the boundary of current cluster. Since a significant part of computational time of DBSCAN is spent for the region queries, BD-DBSCAN focuses on this problem so that the improved version chooses data objects more intelligently for region queries. Also, being a novel idea in the literature of density-based clustering, this approach introduces the notion of a cluster boundary, which is exploited in the selection of influential points –the term *point* corresponds to an individual data object in a dataset, such as a pixel in an image. The improvement reported in [21] saves a huge number of queries (20% - 39% in virtual slides) when compared to DBSCAN, in which one neighborhood query fired for each of points in dataset semi-randomly. 

The idea of BD-DBSCAN in 2D relies on the cluster's boundary, which is a new concept introduced in [21]. Having these borders, we can identify those points that are likely to change the current shape of the cluster’s boundary. Note that the area of a cluster always expands out and never shrinks. In cases where queries cannot affect the cluster's area, the current region query is considered as unnecessary and omitted to fasten the regular DBSCAN. This pre-verification is very helpful in keeping the running time of the whole algorithm low. The intuition behind determining the border of a cluster is derived from the border of a primitive cluster. When boundaries of region queries are united, the outer boundary of this process gives the boundary of the current cluster. Simply, line segments connecting inner and outer boundaries (not only outer boundaries because of the donut problem) are exploited to indicate the border for a cluster. The concept of a convex hull is used so as to i) construct the initial boundary of a cluster and ii) expand the current cluster.

### Initialization of cluster boundary

**Figure 10:**
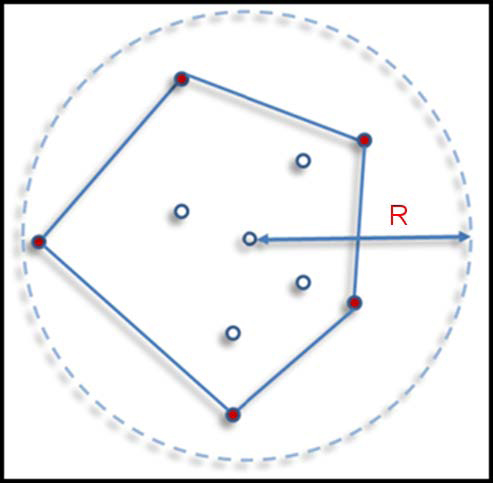
**A primitive cluster as convex hull.** In the forming of a larger cluster, the first step is to initiate the cluster with a polygon, which is a convex hull. This is also defines boundary of an R-neighborhood region. The dashed circled is search space for the query. However, to represent those points, a region of actual points (pixels in an image) is given by convex hull around all points in the dashed circles.

BD-DBSCAN requires two parameters only: *R*, which calibrates how far neighborhood search goes away from a query point; and *MP*, minimum number of points expected in the *R* to form a cluster. *MP* can be also seen as a density parameter in the framework of BD-DBSCAN. The *R* is the radius of the circle in a 2D search space, such as images. We start with an arbitrary pixel *p^A^* of the image. Having a set of all pixels *S^o^* from region query for *p^A^*, we check first if |*S^o^*| is greater than or equal to *MP*. If this condition is met, a convex hull is drawn around *S^o^*. In Figure 10, we depict an example of a primitive cluster consisting of 10 points. 

### Selecting leading points

**Figure 11:**
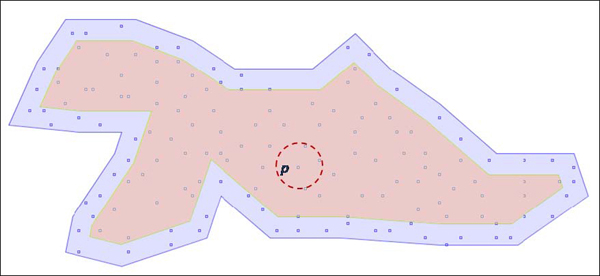
**Leading points (in blue region) can change the shape of a simple cluster, which have no hole in it.** The idea of BD-DBSCAN lies in firing regions queries for those points that are in the blue regions. At a given time, the points in the red regions have no effect in altering boundary of the cluster.

BD-DBSCAN mainly differs from DBSCAN in selecting points in order to expand the cluster. Throughout the clustering, the DBSCAN [29] fires an *R* -neighborhood query for each point *p_i_* in a seedlist of a growing cluster regardless of its impact on current contours of the cluster. This means that *R* -neighborhood queries of those points that cannot alter the boundary of a cluster would be a waste of computational power. Obviously, a certain number of queries would make changes on the shape, while others that are relatively far from the boundaries would not, as in the case of point *p* in Figure 11. On the other hand, it is important to note that most of the expansion made by a query is not final; therefore, it is certainly true that these changes will not be seen in the latest structure of the cluster.

BD-DBSCAN fires only those queries that are likely to expand the boundaries of the cluster in an effort to increase the efficiency of the DBSCAN. To select leading points, it uses boundaries of the polygons that delineate the cluster body. In contrast with DBSCAN, BD-DBSCAN does not keep track of the status of the points, such as *core*. The cluster body can be enlarged only via points that are qualified for *R* -neighborhood query. In other words, if a point is close enough to a cluster boundary, we fire an *R* -neighborhood query around it; otherwise, the query will be omitted, i.e., some queries will not alter the shape of a cluster. Hence, we maintain a set of points that are likely to change the boundaries of a cluster at a given time.

### Expansion of clusters

The innovative algorithm BD-DBSCAN principally behaves similar to DBSCAN and tries to enlarge an existing cluster using unprocessed cluster points. However, we need to inspect the next query in terms of not only newly added points, but also its effect on boundaries. Once the first convex hull is formed around an initial point, it becomes the initial boundary of current cluster. Afterwards, each convex hull around a query point is combined with the main body of the cluster. Principally, this operation corresponds to the union of two polygons. Note that the convex hull is also a special case in the domain of the simple polygon. The notion of expanding a cluster is given in the following definition.

***Definition 6.** Let C be a cluster of points bounded by polygon(s) P*_1_, *P*_2_ … *P_i_* (*i* > 0),* and let P*_1_* be outer polygon for C, i.e., P*_2_ … *P_i_* (*i* > 1)* are to show the holes in P*_1_.* Let T be a newly found convex hull to be merged to main body of a cluster C. Just after merging with convex hull T, cluster C is formulized by C* = (*P*_1_ ∪ *T*)−(*P*_2_ ∪ *P*_3_ … ∪*P_i_*).

Definition* 6* carefully considers the donut problem and uses P_2_ ∪ P_3_ …  ∪P_i_ to exclude these regions from C, shown primarily with P_1_.

**Figure 12:**
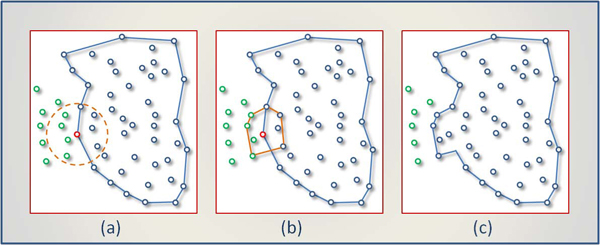
**Expanding a cluster in three steps.** The expanding is basically union of convex hull and current boundary of the cluster.

Adding a small area can expand the cluster in various ways. Figure 12 shows how a newly found convex hull joins the main body of a cluster in three steps. The *R* -neighborhood query (dashed line) in Figure 12(a) for the red point satisfies the *MP* condition; thus, four new points will be added into the existing cluster. The edges of the primitive cluster around the red point, the convex hull in Figure 12(b), change the boundary of the current cluster by merging with it. The final appearance of the cluster's boundary is indicated in Figure 12(c). The expansion of cluster iteratively continues by examining other points in the region of leading points until no more unlabeled point is found for the current cluster. The points that are not associated with any cluster are labeled as noise, as is in DBSCAN.

## Future study

In the future, we will elaborate on ACM to make it more robust against noisy images. Regarding the density-based method, we will focus more on intra-variability and post-assessment during the performance analysis of the intelligent systems. Additionally, the performance of BD-DBSCAN will be evaluated over different polygon-union algorithms.

## Competing interests

The authors declare that they have no competing interests in regards to this study.

## Authors' contributions

MM and NMS have conceived the study. Both of the authors participated in the overall design of the study. MM designed the density-based algorithms. MM developed the general comparison testbed, performed data analysis, algorithm testing, statistical measurements, and benchmarking. NMS designed ACM, integrated it to the general framework, and designed the mask used in the preprocessing step of ACM. Both authors contributed to the writing of this manuscript. Both authors read and approved the final manuscript.

## Abbreviations

ACM: Active Contour model; BD-DBSCAN: Boundary driven density based clustering algorithm with noise; HSL: Hue, Saturation, Lightness; CIE L*u*v: International Commission on Illumination (CIE) color model (1976); PCT: Principal component transform; R: Algorithm parameter, Radius of search region in BD-DBSCAN; MP: Algorithm parameter, Number of minimum points in BD-DBSCAN; A: Parameter in Eq. 1; B: Parameter in Eq. 1; C: Parameter in Eq. 1; s: Parameter in Eq. 3 and Eq. 4, the arc length parameterization of the initial contour; P: Parameter having affect on parameter s in Eq. 3 and Eq. 4; t: Parameter of time step; FP: False Positive; FN: False Negative; TP: True Positive; FN: False Negative; ACHM: Active Convex Hull Model; p^A^: An arbitrary pixel in image; S^o^: Set of all pixel found in region query; C: Cluster of points; P_n_: Polygon; T: Convex hull.

## References

[B1] Jemal A Siegel R Ward E Hao Y Xu J Murray T Thun MJ Cancer statistics. CA: A Cancer Journal for Clinicians 2008 59 225 249 10.3322/caac.20006 19474385

[B2] Argenziano G Soyer HP De Giorgi V Dermoscopy: A Tutorial. 2002 Milan: Edra Medical Publishing & New Media

[B3] Steiner K Binder M Schemper M Wolff K Pehamberger H Statistical evaluation of epiluminescence dermoscopy criteria for melanocytic pigmented lesions. Journal of the American Academy of Dermatology 1993 29 581 588 10.1016/0190-9622(93)70225-I 8408794

[B4] Binder M Schwarz M Winkler A Steiner A Kaider A Wolff K Pehamberger H Epiluminescence microscopy: a useful tool for the diagnosis of pigmented skin lesions for formally trained dermatologists. Archives of Dermatology 1995 131 286 291 10.1001/archderm.131.3.286 7887657

[B5] Fleming MG Steger C Zhang J Gao J Cognetta AB Pollak I Dyer CR Techniques for a structural analysis of dermatoscopic imagery. Comput Med Imaging Graph 1998 22 375 389 10.1016/S0895-6111(98)00048-2 9890182

[B6] Celebi EM Aslandogan AY Stoecker WV Iyatomi H Oka H Chen X Unsupervised Border Detection in Dermoscopy Images. Skin Res Technol 2007 13 454 462 10.1111/j.1600-0846.2007.00251.x 17908199 PMC3191533

[B7] Celebi ME Iyatomi H Schaefer G William Stoecker V Lesion Border Detection in Dermoscopy Images. Computerized Medical Imaging and Graphics 2009 33 2 148 153 10.1016/j.compmedimag.2008.11.002 19121917 PMC2671195

[B8] M. Emre Celebi Hitoshi Iyatomi Stoecker WV Moss RH Rabinovitz HS Argenziano G Soyer HP Automatic Detection of Blue-White Veil and Related Structures in Dermoscopy Images. Computerized Medical Imaging and Graphics 2008 32 670 677 10.1016/j.compmedimag.2008.08.003 18804955 PMC3160648

[B9] Hance GA Umbaugh SE Moss RH Stoecker WV Unsupervised color image segmentation with application to skin tumor borders. IEEE Engineering in Medicine and Biology 1996 15 104 111 10.1109/51.482850

[B10] Carlotto MJ Histogram Analysis Using a Scale-Space Approach. IEEE Transactions on Pattern Analysis 1987 1 121 129 10.1109/TPAMI.1987.4767877 21869382

[B11] Gutkowicz-Krusin D Elbaum M Szwaykowski P Kopf AW Can early malignant melanoma be differentiated from atypical melanocytic nevus by in vivo techniques? Skin Research and Technology 1997 3 15 22 10.1111/j.1600-0846.1997.tb00154.x 27333168

[B12] Gao J Zhang J Fleming MG Pollak I Cognetta AB Segmentation of dermatoscopic images by stabilized inverse diffusion equations. Proceedings of IEEE Int. Conf. on Image Process 1998 823 827

[B13] Dhawan AP Sicsu A Segmentation of images of skin lesions using color and texture information of surface pigmentation. Comput Med Imaging Graph 1992 16 163 177 10.1016/0895-6111(92)90071-G 1623492

[B14] Meas-Yedid V Tilie S Olivo-Marin J Color Image Segmentation Based on Markov Random Field Clustering for Histological Image Analysis. Proceedings of 16th International Conference on Pattern Recognition 2002 796 799

[B15] Schmid P Segmentation of digitized dermoscopic images by two-dimensional color clustering: IEEE Trans Med Imaging 1999 18 164 171 10.1109/42.759124 10232673

[B16] Kass M Witkin A Terzopoulos D Snakes: Active Contour Models. Inter. J. Computer Vision 1987 1 211 221

[B17] Osher S Sethian JA Fronts Propagating with Curvature Dependent Speed: Algorithms Based on Hamilton-Jacobi Formulations. J Comp. Physics 1988 79 12 49 10.1016/0021-9991(88)90002-2

[B18] Acton ST Ray N Biomedical Image Analysis: Tracking. 2006 Arlington, Virginia: Morgan and Claypool Publishers

[B19] Lee I Estivill-Castro V Polygonization of point clusters through cluster boundary extraction for geographical data mining. Proceedings of the 10th International Symposium on Spatial Data Handling 2002 Ottawa, Canada 27 40

[B20] Nosovskiy GV Liu D Sourina O Automatic clustering and boundary detection algorithm based on adaptive influence function. Pattern Recognition 2008 41 2757 2776 10.1016/j.patcog.2008.01.021

[B21] Mete M Delineation of Malignant Areas in Histological Images of Head and Neck Cancer. 2008 University of Arkansas at Little Rock, Applied Science Department PhD thesis.

[B22] Levkowitz H Herman GT A Generalized Lightness, Hue, and Saturation Color Model. Graphical Models and Image Processing 1993 55 271 285 10.1006/gmip.1993.1019

[B23] Ridler T Calvard S Picture thresholding using an iterative selection method. IEEE Trans. Systems Man Cybernet 1978 8 630 632 10.1109/TSMC.1978.4310039

[B24] Celebi EM Kingravi HA Iyatomi H Aslandogan AY Stoecker WV Moss RH Malters JM Grichnik JM Marghoob AA Rabinovitz HS Menzies SW Border Detection in Dermoscopy Images Using Statistical Region Merging. Skin Res Technol 2008 14 347 353 10.1111/j.1600-0846.2008.00301.x 19159382 PMC3160669

[B25] Lilliefors H On the Kolmogorov–Smirnov test for normality with mean and variance unknown, Journal of the American Statistical Association 1967 62 399 402 10.2307/2283970

[B26] Gonzalez RC Wood RE Digital Image Processing, 3rd Edition. 2008 Upper Saddle River, NJ: Pearson Prentice Hall

[B27] Sirakov NM Ushkala K G. Bebis An Integral Active Contour Model for Convex Hull and Boundary Extraction, 2009 Springer 1031 1040 LNCS 5876

[B28] Sirakov NM A New Active Convex Hull Model For Image Regions. Journal of Mathematical Imaging and Vision 2006 26 309 325 10.1007/s10851-006-9004-6

[B29] Ester M Kriegel HP Sander J Xu X A density-based algorithm for discovering clusters in large spatial databases with noise. Proceedings of 2nd International Conference on Knowledge Discovery and Data Mining 1996 226 231

